# Investigation of post-trial access views among study participants and stakeholders using photovoice and semistructured interviews

**DOI:** 10.1136/medethics-2020-107011

**Published:** 2021-06-25

**Authors:** Nothando Ngwenya, Collins Iwuji, Nabeel Petersen, Nompilo Myeni, Samukelisiwe Nxumalo, Ursula Ngema, Janet Seeley

**Affiliations:** 1 Africa Health Research Institute, KwaZulu-Natal, South Africa; 2 School of Nursing and Public Health, College of Health Sciences, University of KwaZulu-Natal, Durban, South Africa; 3 Department of Global Health and Infection, Brighton and Sussex Medical School, Brighton, UK; 4 Interfer, Cape Town, South Africa; 5 Department of Global Health and Development, London School of Hygiene and Tropical Medicine, London, UK

**Keywords:** clinical trials, ethics, HIV infection and AIDS, public health ethics, research ethics

## Abstract

**Purpose:**

We examine the levels of post-trial responsibility ascribed to different stakeholders, following a community-based clinical trial and how the ‘responsibility’ is understood.

**Methods:**

We employed photovoice, unstructured observations and key informant interviews to gain insights into contexts of access to care following transition to the public health system post trial. We used an inductive narrative analysis to explore experiences and understandings of post-trial access (PTA).

**Results:**

In their photovoice stories, many participants expressed a sense of abandonment after the trial. This was viewed as a contributing factor to failing to re-engage with care available in the public health system. This led to the experiences of loss as some trial participants defaulted and died. Research investigators, department of health participants and sponsor agreed that PTA was especially important for communities in resource-limited settings. The government has an obligation towards its citizens while researchers have a responsibility to ensure a smooth transition of patients to public clinics. Sponsors have a responsibility to ensure that the trial is conducted in accordance with the protocol and post-trial agreements are in place and adhered to. Research partnerships among stakeholders were affected by power imbalances making it difficult to negotiate and plan for post-trial care responsibilities.

**Conclusions:**

The research community still struggles with understanding the scope of PTA responsibilities. Power dynamics between public health actors and research sponsors need to be managed to ensure that government involvement is not tokenistic. The responsibility of trial participants and ethics committees needs to be investigated further.

## Background

In 2000, post-trial access (PTA) was included in the Declaration of Helsinki as an issue of concern for researchers, recognising the need to protect trial participants beyond their participation in clinical research. In October 2013, the World Medical Association approved a version of the Declaration of Helsinki which for the first time in the 49 years of its existence requests governments to take responsibility for research participants and their communities.^1^ There is still much debate within the research community on the meaning and interpretation of the amendment. This 2013 Declaration of Helsinki amendment states that, ‘at the conclusion of a study, patients should be assured of access to the best proven prophylactic, diagnostic and therapeutic methods identified by the study’; the 2006 Universal Declaration on Bioethics and Human Rights goes further suggesting that PTA should be more than just the availability of the investigated medicine.^2^ The United States National Bioethics Advisory Committee recommends that sponsors and researchers make good faith efforts in ensuring continued access to experimental interventions that have been proven effective for the participants.^3^ Yet, these statements do not offer guidance on how this can and should be done.^4–6^

Focusing on an investigational drug or intervention for PTA omits the equally important aspects of the care of a participant that goes beyond the drug.^7 8^ Other responsibilities for post-trial care include arranging the transition to other clinical care, ensuring existence of appropriate follow-up and arranging social support services to ensure a smooth transition to arranged clinical care.^9 10^

Ethical responsibilities of researchers, sponsors and even the government that are concerned with the health and safety of participants are usually laid out clearly during the trial and yet these abruptly come to an end with trial completion.^9 11^ This is of critical importance in many low-income and middle-income countries where participants may not have access to affordable healthcare systems leading to potential exploitation of vulnerable communities.^4 8 10^ The challenges associated with PTA of drugs or care comprise of issues including distributive justice, potential for exploitation and the argument to treat participants with human dignity.^12 13^

In this article, we examine how the meaning of responsibility is understood and levels ascribed to different stakeholders in a study using a Universal Test and Treat (UTT) HIV trial as a case study.^14^

## Methods

We integrated photovoice, unstructured observations and semistructured interviews. We began by identifying PTA principles in international legislation and guidelines and reviewed current academic literature to develop the topic guide. Photovoice is a visual participatory approach that involves participants taking photos to help them document and narrate their story around certain issues of concern while promoting critical dialogue. It was appropriate for this study as we sought to empower individuals to ‘voice’ their individual perceptions and experiences of PTA to treatment and any impact on their lives and photovoice engages with participants from marginalised communities in identifying issues that affect them from their own point of view.^15^

### Study setting

This study was undertaken in the rural Hlabisa subdistrict of uMkhanyakude district, KwaZulu-Natal province, South Africa. uMkhanyakude is one of the poorest districts in South Africa; only about 22% of the population have access to piped water and sanitation.^16^ The area is characterised by dispersed rural settlements with most households depending on small-scale agriculture and government grants. Outmigration to urban centres for work is quite common. Despite the large scale up of HIV care and treatment in South Africa, the area still experiences the highest HIV incidence among adolescent girls and women and has a high HIV burden.^16 17^

### Description of case study

This research is based on a case study of a UTT trial that used antiretroviral treatment (ART) as a prevention method to decrease the risk of HIV transmission implemented in purpose-built trial clinics from 2012 to 2016. The main eligibility was that the individual was HIV-positive. The trial provided comprehensive care for HIV-positive individuals including care for common medical conditions or any intercurrent illness not requiring hospital admission. The primary outcome of the trial was a measure of HIV incidence, using longitudinal dried blood spots from participants every 6 months. Prior to trial completion, other evidence from NIH-sponsored START trial and the ANRS-sponsored TEMPRANO trial reported that early ART initiation has significantly greater health benefit to HIV-positive people.^18 19^ Given this effectiveness of ART initiation outside standard practice, consideration of PTA would be essential for patients’ well-being. Another concern for PTA in this study was the provision of 22 new study clinics located close to people’s homes in an area with only three public clinics. During the informed consent process and at various intervals during the trial, participants were informed that after the completion of the trial or if they decided to withdraw, they would be transferred to the local department of health clinics’ treatment and care programme where they would continue to receive HIV care and treatment. During the last year of the trial, participants were informed of the transition procedure and that their records will be transferred to their preferred government clinic.

### Current study

**Figure 1:**
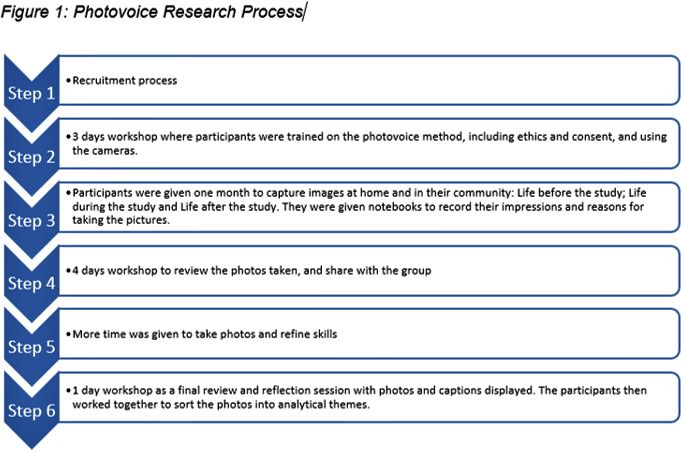
Photovoice research process.

Photovoice was used as a visual participatory method to engage with participants in a series of training and discussion workshops. These three workshops took place over 8 days (figure 1), facilitated by NP, an expert in community engagement using participatory methods. A convenience sample of participants that had been involved in the UTT trial and their families was identified through the Africa Health Research Institute database and invited according to the following categories: (1) families with a member who is still adhering to treatment at the clinic, (2) families with a member who is no longer adhering to treatment at the clinic and (3) families with a member who is deceased. Recruitment for this study took place over 2 weeks in January 2019. Overall, 10 out of 12 people invited (3 male, 7 female) enrolled in the photovoice project.

The inception workshop took place over 3 days where participants had comprehensive training on the methods, on using the cameras and ethics of photovoice. They had the opportunity to take pictures and receive guidance. They then had 1 month to capture images within their community that illustrated their perception of life before the trial, life during the trial and life after the trial. They were given notebooks with specific prompts: (1) Who or what is in the photo? (2) When did you take it? (3) Why did you take the picture? and (4) How does it make you feel? This was later used by the participants to construct their narratives and contextualisation for each specific photograph. All photographs were developed and printed in colour during the next workshop which lasted 4 days. During this workshop, participants reviewed their pictures and shared as a group why they took the picture. We applied narrative analysis techniques to explore the photographs and frame its accompanying narration and stories, as led by the participants.^20 21^ The participants led the categorisation and thematic grouping of photos and discussions. Some people asked for more time to take more pictures as they felt that they had not followed the brief. This was followed by the final review and reflection day where they discussed their photographs and the researchers assisted by typing out the narration/caption of each photograph. The same methodological process using narrative analysis techniques was applied to these photos. These were displayed on a wall and participants then organised the photos thematically and by prioritisation. On this last day, participants were invited to sign a release form for their photos and videos taken. Permission for reuse of photos was obtained from all participants. Participant identifiers are not used for photos or quotations to protect the privacy of the research participants.

Semistructured interviews were conducted with principal investigators (PIs) of the trial, members of the subdistrict, district and provincial level Department of Health (DoH) and a sponsor between October 2019 and February 2020. Purposive sampling was employed with an invitation sent to the PIs and members of DoH who were aware of and at some point, involved in the trial. The goal of the interviews with PIs, DoH and sponsor was to explore the pretrial agreements, what happened after the trial, whether this was in line with PTA guidelines and protocol and ideally what the different responsibilities of each of these stakeholders should be. All the interviews were conducted by the first author (NN) working with two of the coauthors (SN and UN) who facilitated data collection and another coauthor who is a public engagement officer (NM). Preliminary analysis was conducted manually by three of the authors using open coding, guided by the study research focus but also following a grounded theory approach to capture new themes emerging from the data. Following manual coding, the data were uploaded and recoded using N-Vivo V.12 software and analysed thematically. Emerging themes were discussed and compared across the dataset. The recurrent pattern of these new themes gave confidence that key areas had not been omitted from the dataset.

## Results

We present the visual narratives from the photovoice part of the study as well as extracts from the in-depth interviews regarding perceptions of PTA responsibilities and community needs. Two interviews were conducted by telephone and four were conducted face to face. Community needs (lack of care access, tradition and support, loss and desertion) is one of the major themes abstracted from the participant-participatory analysis while the researchers and DoH professionals focused on expressing their understanding of the different responsibilities that all stakeholders should have. The visual narratives aim to demonstrate the community’s views on the impact of the trial as well as the ending of the trial in relation to access to care.

Participants took over 120 photographs during the entire project which were then categorised into 8 categories. These categories were further organised around three themes: (1) lack of access, (2) tradition and support and (3) loss and desertion.

### Lack of access

**Figure 2:**
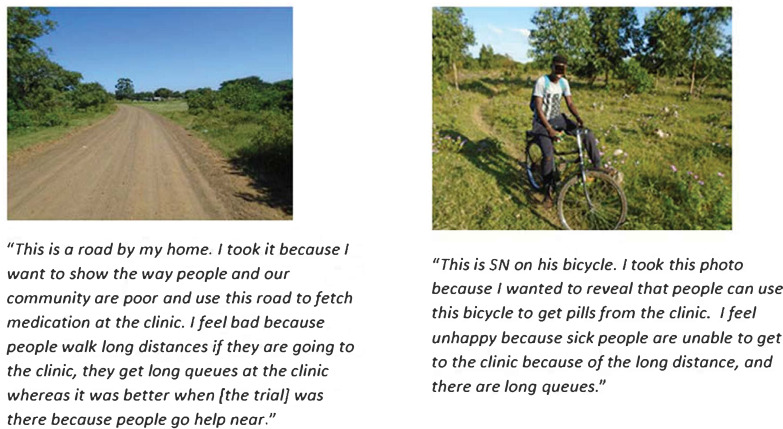
(A, B) Lack of access.

Participants expressed how access to healthcare services was quite problematic and they had to either walk a long distance to get to their clinics for access to treatment or find other means of transport which is not easily available to everyone as illustrated in figure 2A, B. The concept of removing the trial facilities that offered ARTs close to their homes was a topic of discussion and participants felt that they were neglected by the research institute. They emphasised how the trial clinics close to their homes was part of the intervention as much as the ARTs were.

### Tradition and support

**Figure 3:**
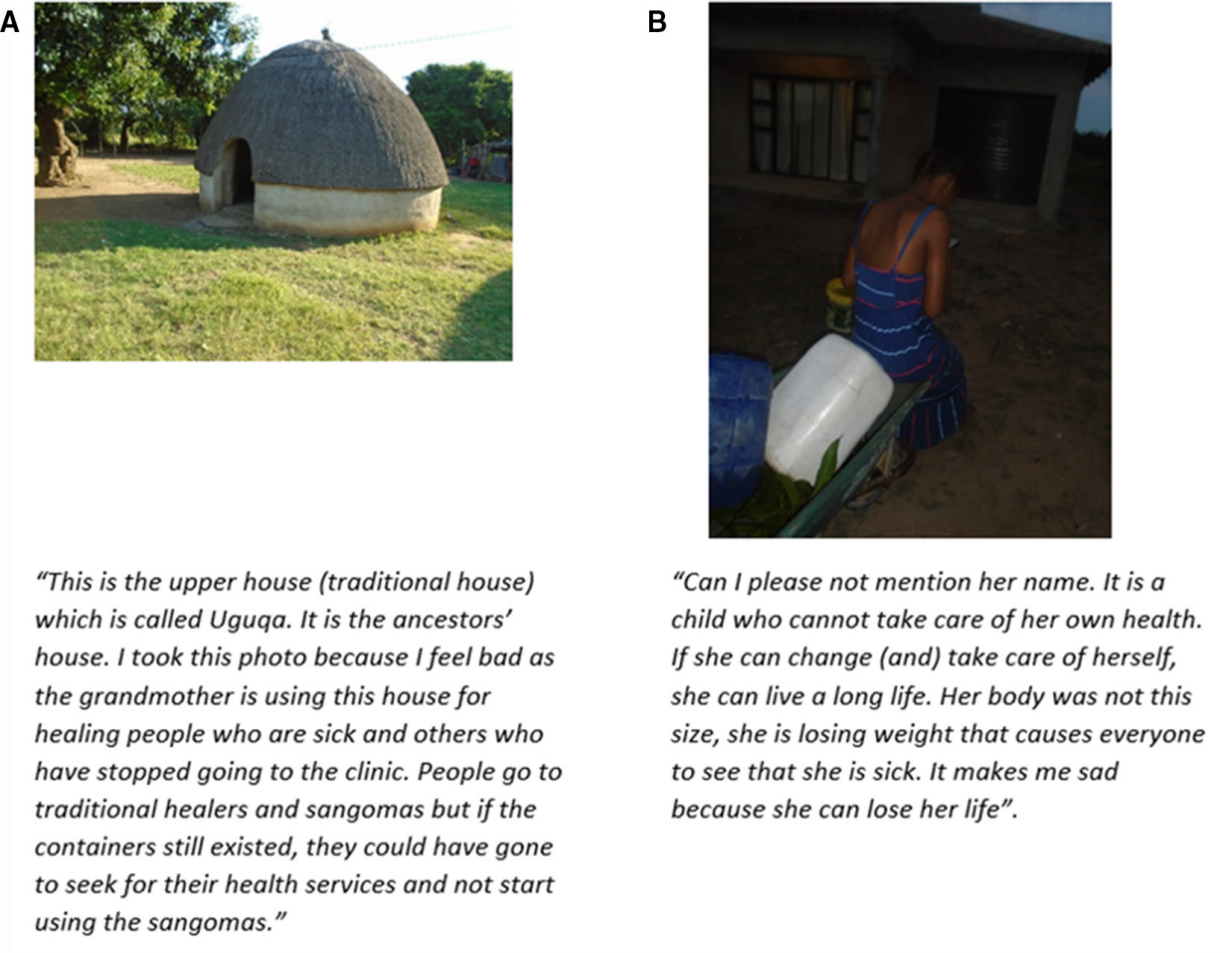
(A, B) Tradition and support.

Throughout the project, narratives were shared that showed the impact of post-trial care as portrayed in figure 3A, B. All the participants shared how the community members recognised the significance of the end of the trial as well as observed the impact of this through people resorting to traditional medicines and defaulting from treatment.

### Loss and desertion

**Figure 4:**
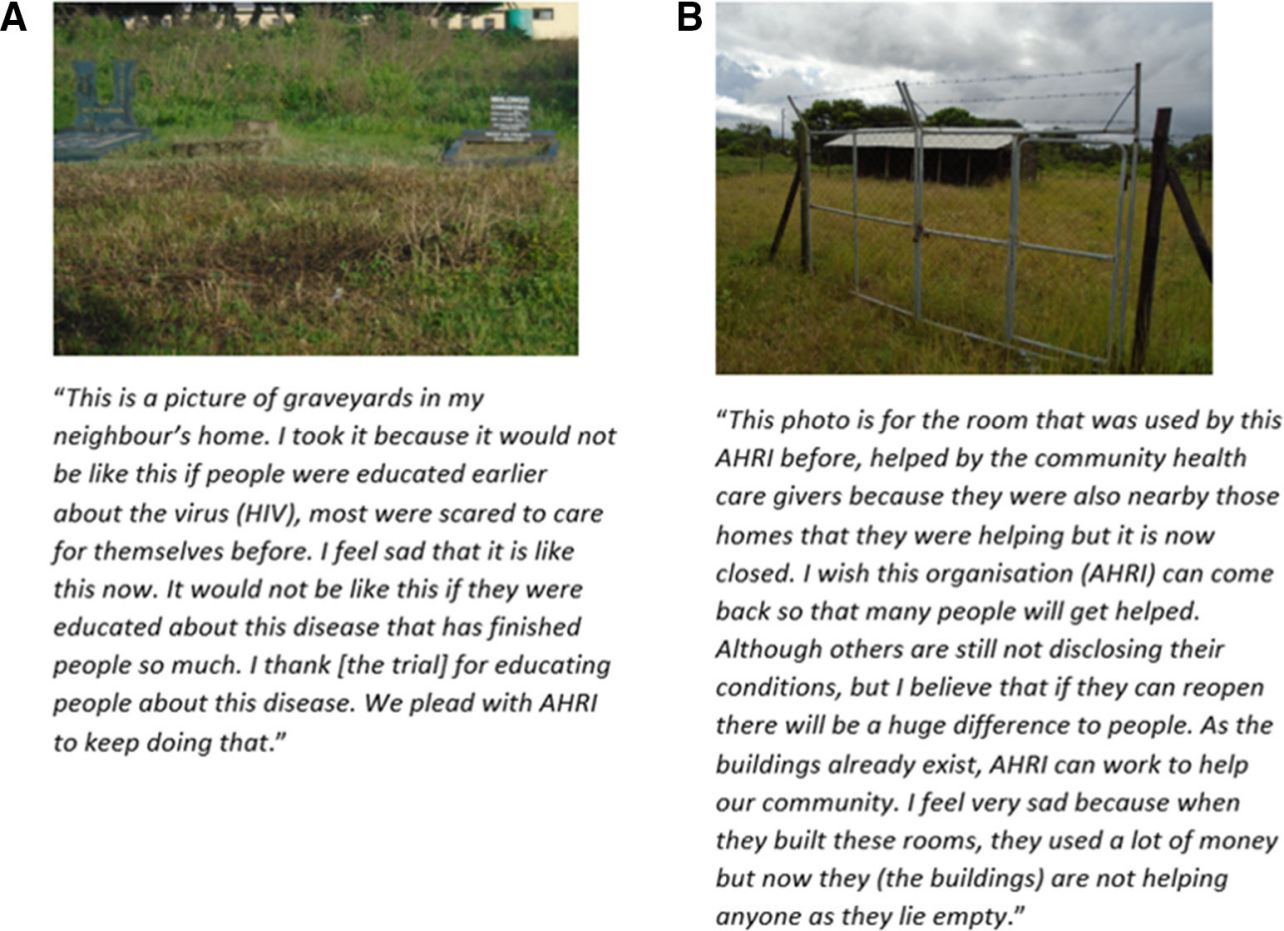
(A, B) Roles and responsibility.

The perception of the trial being linked to loss permeated some of the narratives which highlighted the emotional responses to the death of loved ones and other villagers as shown in figure 4A, B. Participants also shared how the deserted clinical trial facilities were a constant reminder of life post trial and emphasised the need for AHRI to reinstate this trial which many viewed as a service rather than research.

### Roles and responsibilities from interview participants

#### Legal obligation to provide PTA for the government

Participants shared their perspectives of the different responsibilities that stakeholders had and to whom they had that responsibility. Responsibilities were described as giving access to treatment and sometimes as taking care of participants as one PI said, ‘what had been agreed prior to the trial starting, …was that the Department of Health had agreed to make treatment available to all participants at the end of the trial’. This responsibility was also shared by individuals from the department of health as one participant said, ‘So, it should be the Department of Health that would eventually take over’. Sentiments were however shared on the financial challenges for the government due to limited resources, *‘*Department has to look at the numbers of patients on treatment and then develop the way forward like we’d have to see whether we would be able to cater if we had enough budget’. One DoH interviewee shared sentiments on power dynamics between researchers and the government that could potentially lead to pressurised agreement of PTA as the government needs to look after its people, ‘In most trials there is always a delay because DoH has not signed because DoH is concerned about this and that and that and that. However, the pressure, you know academic people have…So most of the time, DoH tries to look after the interest of the public. But the odds are usually against DoH. Somebody ends up signing under pressure’.

#### Responsibility of awareness of PTA for sponsors

There were mixed views when it came to the responsibility of the sponsors. Two investigators’ perspectives were that the sponsor did not have a direct responsibility for PTA. Research staff expressed that the sponsor had ‘a responsibility or an awareness (of PTA) and that they should also you know, be receptive to further applications, I guess, for support relating to questions that come out of the trial that they have sponsored. I do not think that the sponsors have got a direct responsibility for post-trial access’. The sponsor interviewee however shared that the sponsor had a legal responsibility that any agreements documented in a protocol are carried out and expressed that ‘My view is that the sponsor is responsible for all aspects that the trial should take place under perfect conditions according to all the rules that exist…and that includes the post-trial provision’. This person referred us to the sponsor’s ethics charter for research in developing countries which states that part of their requirement from investigators when applying for funds is to ‘define postresearch support conditions’ and to discuss the provision of equivalent interventions if effective treatment/interventions are not available at the end of the project.

One DoH participant seemed to think that sponsors had a bigger responsibility than shared by others and contrasted policy or legal responsibilities of the sponsor to the mining industry where mines have a legal responsibility to rehabilitate the environment after mining. Research is a ‘data mining operation’ and should have a policy that requires a form of rehabilitation of communities and the sponsor should have a financial responsibility for rehabilitation. They went on to explain that better provision of PTA and/or responsible transition of research participants is needed to ensure that they are not left in a worse condition than they were before the trial.

#### Social responsibility by investigators

There seemed to be general agreement from the three PIs that they had some responsibility to ensure benefit to the community and to avoid harm to participants, ‘I think the responsibility is to ensure that trial participants or those willing to enter a trial on behalf of research, benefit themselves and that based on the outcome of the trial, of the study, that the broader community benefits from that’. Investigators shared how their responsibility was mostly to ensure no harm came to the participant during the trial and part of that was to support the transition to the public HIV treatment programme, as one said, ‘it is not the responsibility of the scientists to organize the HIV treatment program thereafter, but it is the responsibility of the science to make sure that that could happen by national program and to support that’.

Two other stakeholders who had not been considered prior the study were raised by the interviewees; the ethics committee and its responsibility to monitor how investigators consider PTA in the protocol and the participants themselves as one interviewee said, *‘*responsibility lies with the individual who participated voluntarily in the study’, as they would have been given all the information including what would happen at the end of the trial before the trial commenced. PIs however felt that this was a challenge as one said, ‘there’s a very poor understanding of research, internationally there’s a poor understanding of research by research participants within (research Institute) research participants’ and therefore this would make it difficult to place responsibility with the community members. One researcher echoed and offered an explanation for the poor understanding and expressed that the nature of the trial was ‘less of a clinical trial and more as part of a service delivery so I can understand that’s what the community sees as well,… it was a clinical trial but it was more of an implementation trial’ which also had its own implications as the institute functioned as a service provider which was providing specialised care to individuals.

## Discussion

Similar to previous studies, our findings reveal the extent of the impact of PTA including concerns of abandonment and potential for defaulting treatment.^4 8^ The practicalities are quite challenging and not as easy to implement as stakeholders involved cannot individually guarantee PTA due to many other considerations such as costs and feasibility.^1 5 7^ The UTT trial participants shared feelings of abandonment after the trial closure leading to their reliance on traditional medicine and culture for support. One explanation of this could be the lack of understanding of research compared with healthcare service provision with participants seeing the trial as a service to them that would not come to an end.

The study illustrated how factors ascribed to responsibility are inter-related with challenges in conceptualisation and understanding of responsibility sometimes leading to major flaws in practice. Most researchers perceive that the government has a legal obligation as the National Department of Health to fulfil its mission to improve the health and healthcare delivery systems for its citizens. This finding supports the DoH amendment to include governments as a stakeholder that has a responsibility for PTA to ensure continued access to treatment for its people.^22 23^ However, the power dynamics expressed by the DoH interviewees have implications on the trial agreements drawn at the beginning of the trial. Therefore, mechanisms are needed to mitigate differentials in power if these trial agreements are to be used as instruments to implement PTA obligations.

In normative discussions, the investigators have a social responsibility as ‘scientists’ which often is driven by the usefulness of the research outcomes and addressing relevant health problems.^24 25^ Social responsibility is not about an individual investigator, but is an ethical aspect that is operational within an organisation and governs its conduct.^26^ Socially responsible and responsive health research practices focus on doing no harm which would involve responsible transitioning of participants to the standard of healthcare they need so as to leave people no worse off than before the trial.^9^ On the other hand, the sponsor has a legal responsibility according to Good Clinical Practice guidelines to ensure and monitor that the trial is conducted according to the protocol including ensuring that any post-trial agreements are in place and adhered to. What remains unanswered by this study is what is considered a reasonable execution of this responsibility from the various stakeholders.

An interesting finding is the question raised on the responsibilities of ethics committees and trial participants. Further research is needed to explore the responsibilities of these stakeholders as raised in this study.

Our exploratory analysis is subject to certain limitations including the modest sample size due to our access to participants and as such the conclusions are not definitive. There is great potential for recall bias due to the duration between the end of the case study trial and the current study. Generalisation of the findings to other study trial may be limited, because in the case study trial, the drugs were freely available in the public ART programme, however the study highlights that aspects of ‘care’ extend beyond a narrow conception of access where the availability of a drug free of cost was not a barrier, as participants expressed negative experiences because of the end of the trial. The triangulation of data collected add value to the project and photovoice empowered participants to decide the narrative of importance to them on their PTA experiences and concerns held. Future research could include an exploration of these post-trial aspects in the other UTT trial sites for comparability and cross-fertilisation lessons.

## Conclusion

The issue of PTA to treatment is an ethical dilemma that is still debated and the research community struggles with understanding the scope and extent of responsibilities. Implications of the implementation of PTA guidelines is challenging due to these differing views and understanding of responsibilities among different stakeholders, and this study shows how some participants are bearing the burden of this uncertainty. This small exploratory study highlights voices of participants in their experience of lack of access, loss of support and desertion. It further identifies an additional implementation challenge of power dynamics between public health actors and research sponsors, suggesting that there is a significant gap between ensuring continued availability of trial treatments and maintaining a sense of care for the participants.

## Data Availability

Data are available upon request.
